# Dynamic time warping outperforms Pearson correlation in detecting atypical functional connectivity in autism spectrum disorders

**DOI:** 10.1016/j.neuroimage.2020.117383

**Published:** 2020-09-17

**Authors:** A.C. Linke, L.E. Mash, C.H. Fong, M.K. Kinnear, J.S. Kohli, M. Wilkinson, R. Tung, R.J. Jao Keehn, R.A. Carper, I. Fishman, R.-.A. Müller

**Affiliations:** a Brain Development Imaging Laboratories, Department of Psychology, San Diego State University, 6363 Alvarado Ct., Suite 200, San Diego, CA 92120, United States; b San Diego State University/University of California San Diego Joint Doctoral Program in Clinical Psychology, San Diego, CA, United States

**Keywords:** Functional MRI, Functional connectivity, Dynamic time warping, Timeseries analysis, Resting state, Autism spectrum disorder

## Abstract

Resting state fMRI (rsfMRI) is frequently used to study brain function, including in clinical populations. Similarity of blood-oxygen-level-dependent (BOLD) fluctuations during rsfMRI between brain regions is thought to reflect intrinsic functional connectivity (FC), potentially due to history of coactivation. To quantify similarity, studies have almost exclusively relied on Pearson correlation, which assumes linearity and can therefore underestimate FC if the hemodynamic response function differs regionally or there is BOLD signal lag between timeseries. Here we show in three cohorts of children, adolescents and adults, with and without autism spectrum disorders (ASDs), that measuring the similarity of BOLD signal fluctuations using non-linear dynamic time warping (DTW) is more robust to global signal regression (GSR), has higher test-retest reliability and is more sensitive to task-related changes in FC. Additionally, when comparing FC between individuals with ASDs and typical controls, more group differences are detected using DTW. DTW estimates are also more related to ASD symptom severity and executive function, while Pearson correlation estimates of FC are more strongly associated with respiration during rsfMRI. Together these findings suggest that non-linear methods such as DTW improve estimation of resting state FC, particularly when studying clinical populations whose hemodynamics or neurovascular coupling may be altered compared to typical controls.

## 1. Introduction

The use of resting state functional magnetic resonance imaging (rsfMRI) has been growing exponentially since it was first introduced by Biswal et al. (1995). Numerous studies have implemented rsfMRI to assess brain function in clinical populations, and in the search for biomarkers of neurodevelopmental and neuropsychiatric disorders (Birn, 2012; Fox and Greicius, 2010; Lee et al., 2013; Leuthardt et al., 2018). Yet, its reliability has been relatively low and findings have been inconsistent (Badhwar et al., 2019; Hull et al., 2017; King et al., 2019; Noble et al., 2019). The rsfMRI method is founded on the observation that similar blood-oxygen-level-dependent (BOLD) signal fluctuations in two or more brain regions measured by fMRI during the resting state reflect a common functional process, i.e. that they are “functionally connected” (Guye et al., 2008; Lee et al., 2013). Coordinated BOLD activity across the brain may reflect a history of co-activation (Dosenbach et al., 2008; Guerra-Carrillo et al., 2014; Lewis et al., 2009). However, the degree to which similarity of BOLD fluctuations is detected largely depends on the method used to quantify this relationship.

**Fig. 1.**
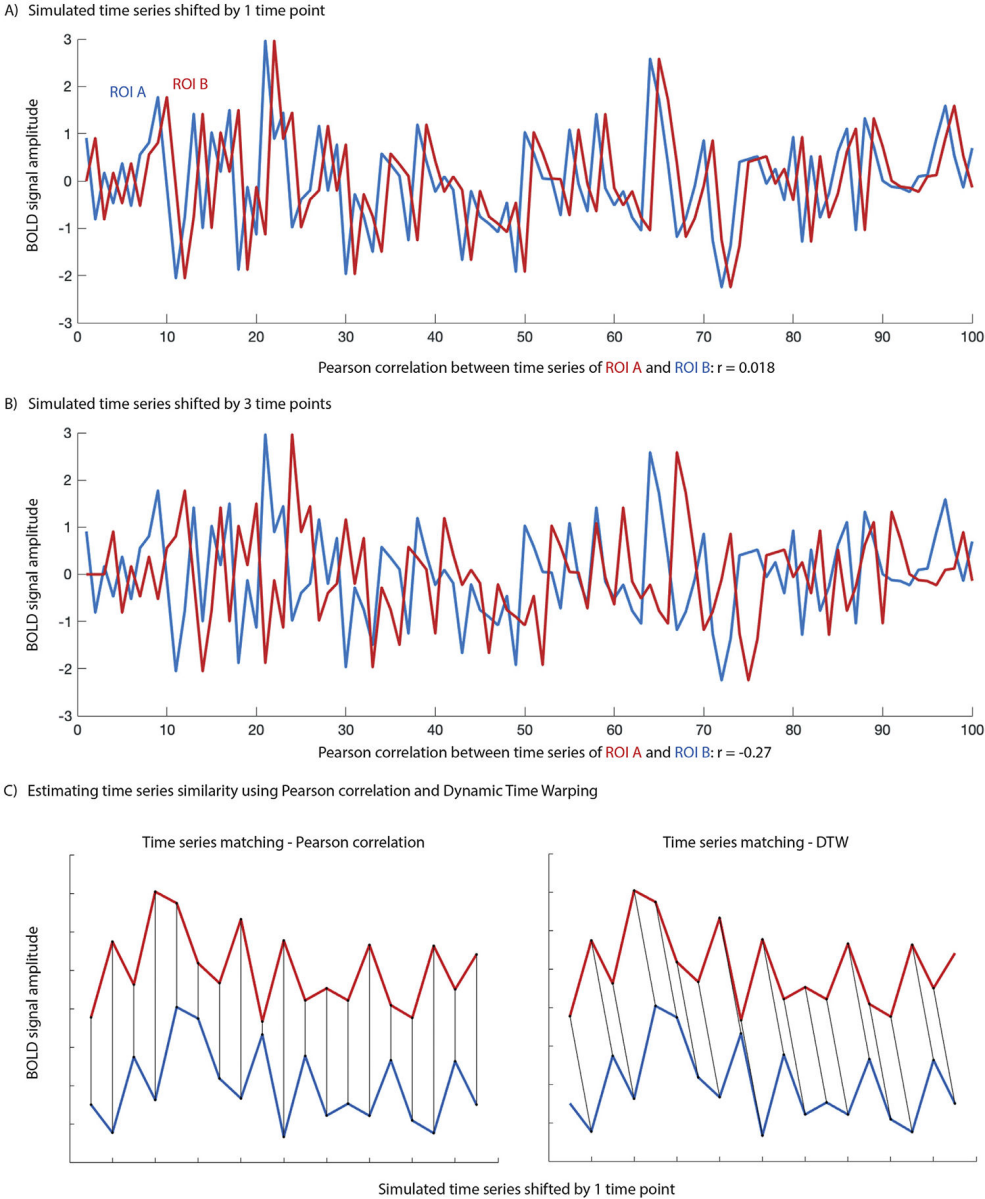
A) Pearson correlation between a simulated time series (ROI A) and the identical time series shifted by 1 time point (ROI B) illustrates the assumption of synchronicity when assessing time series similarity using Pearson correlation. B) A large enough lag between time series will render a Pearson correlation of two otherwise identical time series negative. C) Matching of time series when calculating time series similarity using PC (left) or DTW (right) illustrates how PC might underestimate time series similarity when there is lag.

As in the milestone study by Biswal et al. (1995), resting state functional connectivity (FC) has almost exclusively been estimated using Pearson correlation. Pearson correlation (PC) is a linear method and assumes that for two regions to be deemed functionally connected, BOLD synchronicity is essential. PC does *not* take into account the temporal structure of the signal (such as temporal autocorrelation or possible lag between time series). High synchronicity (and consequently a high Pearson correlation) between two time series suggests that the two regions may be involved in the same process or that the signals share a common source. However, the inverse is incorrect: Low BOLD synchronicity (or even a negative Pearson correlation) does *not* necessarily imply unrelated functional processing and absence of FC. A number of factors can contribute to low or negative FC as estimated using Pearson correlation. The fMRI BOLD signal is a hemodynamic response that arises from neurovascular coupling and is related to vascular anatomy, as well as metabolic, physiological and neurochemical activity. Not surprisingly, hemodynamics have been shown to change with development and aging (Arichi et al., 2012; Asemani et al., 2017; D’Esposito et al., 2003; Huettel et al., 2001; West et al., 2019), and to differ in clinical populations (Ford et al., 2005; Gao et al., 2016; Mark et al., 2015; Mayer et al., 2014; Reynell and Harris, 2013; Yan et al., 2018). The shape and timing of the hemodynamic response also varies across the brain (Chen et al., 2015a; Lindquist et al., 2009), with a lag of approximately −5 s to + 4 s relative to the signal in the superior sagittal sinus (one of the blood vessels clearly visible on MRI, and in which blood flow can be reliably tracked in; Tong and Frederick, 2010). This variability of the hemodynamic response can bias FC estimates derived with Pearson correlations (Rangaprakash et al., 2018). For example, even a small lag between two otherwise identical time series leads to low Pearson correlation and consequently to drastically underestimated time series similarity (Fig. 1A). A large enough lag can even result in negative Pearson correlations (Fig. 1B). If perfect synchronicity is the only requirement to deem BOLD activity in two regions functionally related, Pearson correlation adequately quantifies the degree of similarity or “functional connectivity”. Given known spatial differences in timing and shape of the hemodynamic response, however, it seems unlikely that this would capture the true relationship between neural activity changes in different brain regions.

The insensitivity to time series structure and lag is particularly problematic when studies assign biological meaning to the magnitude of Pearson correlations, as in comparisons of FC across the lifespan, or between clinical populations and healthy controls. A long-standing debate in the rsfMRI autism literature, for example, revolves around whether individuals with autism spectrum disorders (ASDs) show functional over- or underconnectivity (for a review see Hull et al., 2017), with underconnectivity between networks often interpreted as a lack of integration between regions (e.g. Rudie et al., 2012). The impression of lower connectivity sometimes reflects negative FC estimates; however, as illustrated in Fig. 1, even strongly negative Pearson correlations do not necessarily imply reduced similarity between time series. Negative correlations pose an even greater challenge in comparisons with behavioral measures. Such brain-behavior relationships are usually interpreted in purely linear terms. For example, a positive correlation between FC and ASD symptom severity would be summarized as lower FC – often interpreted as reduced integration between regions – being associated with low symptom severity, even if driven by negative Pearson correlations.

Previous studies have shown that methodological differences in rsfMRI preprocessing and denoising alter the distribution of FC estimates and account for some inconsistencies in the literature (He et al., 2019; Nair et al., 2014). Most prominently, global signal regression (GSR) removes the mean BOLD time series across the brain during denoising of rsfMRI data in order to control for global noise (e.g., from respiration, Power et al., 2017a). This shifts the distribution of observed Pearson correlations, rendering estimated FC between some regions negative (Murphy et al., 2009), and exemplifies the challenges of interpreting Pearson correlation magnitude with respect to the presence or absence of “functional connectivity”. Quantifying the similarity of time series and making inferences about the degree to which they reflect a common neural process, requires methods that are sensitive to time series structure and robust to relatively minor changes in data pre-processing and denoising. Thus, functional connectivity estimates might be improved by methods other than Pearson correlation that do not assume linearity, account for time series structure (i.e., are robust to lag and HRF differences), and result in a more easily interpretable measure of time series similarity.

Many alternatives to Pearson correlation exist to characterize the similarity between time series. Some have been proposed for estimating FC from rsfMRI, but none have been widely adopted (Anzellotti and Coutanche, 2018; Chen et al., 2015a,b; DSouza et al., 2018; Gultepe and He, 2013; Liu et al., 2018; Mohanty et al., 2020). Meszlényi et al. (2017b) recently proposed dynamic time warping (DTW) as a more accurate way to quantify BOLD time series similarity, with promising initial results. DTW was first developed to facilitate speech recognition (Sakoe and Chiba, 1978), but has also previously been applied to study similarity of neural responses recorded using electrocorticography (Cho et al., 2004) and to compare responses from electroencephalography and fMRI (Dinov et al., 2016). It is an elastic matching algorithm and as such can account for lag and shape differences between time series, while also being more robust to linearly combined global noise and accounting for temporal autocorrelation. Its output reflects a distance measure of the amount of warping required to align two time series. Using both simulations and repeat rsfMRI acquisitions from one participant, Meszlényi et al. (2017b) found that compared to Pearson correlation estimates of FC, DTW resulted in higher within-subject test-retest reliability and increased robustness to differences in preprocessing and denoising methods, including reduced sensitivity to global noise and GSR. Additionally, they demonstrated in an open-source dataset of 26 participants (14 males) that gender could only be classified from FC patterns derived using DTW, but not Pearson correlation or cross-correlation. In another study utilizing DTW to estimate FC, Meszlényi et al. (2017a) showed that classification of patients with amnestic mild cognitive impairment was improved when multiple different connectome measures were used as features.

Studies of clinical populations may benefit from such non-linear methods, especially when the goal is to identify atypical FC patterns and their relationship to behavior and symptomatology. ASDs are generally characterized by differences in the network organization and communication of the brain (Di Martino et al., 2014; Hull et al., 2017). However, despite decades of research and hundreds of studies investigating atypical functional connectivity in ASDs, there is little consensus regarding the exact nature of these differences, and no clear biomarkers have yet been identified (He et al., 2019; Plitt et al., 2015). Autism encompasses a highly heterogenous spectrum of disorders, and in addition to methodological differences mentioned above, cohort effects likely contribute to inconsistent findings (Lenroot and Yeung, 2013). A mostly neglected factor may be the limitations of Pearson correlation described above, which may prevent reliable identification of atypical functional network organization in ASDs, especially in view of neurophysiological differences affecting neurovascular coupling (for a review see Reynell and Harris, 2013). For example, HRF (Yan et al., 2018) as well as BOLD lag structure (Mitra et al., 2017) have been shown to differ in individuals with ASDs compared to typical controls. Therefore, a method such as DTW that is robust to such differences when estimating FC may be particularly beneficial to the investigation of altered brain function in ASDs and its relation to symptomatology.

In the current study, we first test the impact of motion on DTW and PC estimates of FC and replicate the findings of increased robustness to GSR and higher test-retest reliability of DTW estimates of FC reported by Meszlényi et al. (2017b) (Analyses 1 and 2). We then show that DTW is more sensitive to brain function than PC by comparing FC between resting state and task performance, with predicted changes in FC due to frequent button responses during the task (Analysis 3). Lastly, we assess the sensitivity of DTW compared to PC to detect alterations of FC and relationships between FC, behavior and physiological confounds in individuals with ASDs compared to matched typical controls (Analyses 4 and 5).

## 2. Materials and methods

### 2.1. Participants

Data from three cohorts were analyzed for the current study. Both resting state and task fMRI were collected in an adolescent cohort (*n* = 22, 12–19 years), and resting state fMRI only was collected in a cohort of children and adolescents (*n* = 99, 7–18 years) and in an adult cohort (*n* = 38, 40–60 years). Demographic and clinical data for the three cohorts are summarized in Table 1. Participants were recruited for a study on lexical decision in adolescents with ASD, a study on childhood brain development, and a longitudinal study on ASD in adulthood. Individuals with comorbid ASD-related medical conditions (e.g., Fragile-X syndrome, tuberous sclerosis, epilepsy), or other neurological conditions (e.g., Tourette syndrome) were not eligible for participation. Exclusionary criteria for the typical control (TC) groups were: personal or family history (first degree relatives) of ASDs or other developmental disorders, neurological disorders, and severe mental illness including schizophrenia, bipolar disorder, severe major depressive disorder, and obsessive-compulsive disorder. Informed consent or assent was acquired from all participants or their caregivers, and participants were compensated for their time. All study protocols were approved by the institutional review boards of the University of California San Diego and San Diego State University.

### 2.2. Diagnostic assessments

ASD diagnoses were confirmed based on Diagnostic and Statistical Manual of Mental Disorders 5th edition (DSM-5) criteria (APA, 2013), supported by the Autism Diagnostic Observation Schedule, Second Edition (ADOS-2; Lord, 2012); the Autism Diagnostic Interview-Revised (ADI-R; Rutter et al., 2003) in children and adolescents; and expert clinical judgement.

### 2.3. MRI acquisitions

MRI data were collected at the University of California San Diego (UCSD) Center for Functional MRI (CFMRI) on a GE 3T Discovery MR750 scanner using a Nova Medical 32-channel head coil (adolescent and adult cohort) and an 8-channel head coil (child/adolescent cohort).

#### 2.3.1. Child/Adolescent cohort

A single-shot gradient-recalled EPI sequence (180 whole-brain volumes were acquired (TR = 2000 ms; TE = 30 ms; slice thickness = 3.4 mm; flip angle = 90°; FOV = 22.0 mm; matrix = 64 × 64; in-plane resolution = 3.4 mm^2^) was used to acquire 6 min of resting state fMRI. High-resolution T1-weighted sequences (3D FSPGR; 1 mm isotropic voxel size, NEX=1, TE=min full, TI=600, flip=8°FOV=25.6 cm, matrix=256 × 256, receiver bandwidth 31.25htz) were collected in each participant.

#### 2.3.2. Adolescent cohort

A multi-echo multi-slice (MEMS) echo planar imaging (EPI) sequence allowing simultaneous acquisition of multiple slices at multiple echo times (Kundu et al., 2012; Olafsson et al., 2015), was used for collection of a resting state (6 min) fMRI scan, as well as during the performance of a lexical decision task (2 × 7 min) with the following EPI parameters: TR=1100 ms, TEs=13.2/30.3/47.4 ms, flip angle=60°, FOV=21.6 cm, acquisition matrix=72 × 72, 45 slices, voxel size=3mm ^3^. EPI data from the different echo times were optimally combined and – to keep pre-processing and denoising comparable to more common single echo EPI acquisitions (also see Discussion) – pre-processed using a standard pre-processing and denoising pipeline described below. Since DTW estimates are dependent on the length of the time series, only the first 300 volumes of each run were analyzed. High-resolution structural images were acquired with the same FSPGR T1-weighted sequence as in the child/adolescent cohort.

#### 2.3.3. Adult cohort

A multiband EPI sequence which allows simultaneous acquisition of multiple slices was used to acquire two back-to-back fMRI runs (6-minute duration each) with high spatial and temporal resolution (TR=800 ms, TE=35 ms, flip angle 52°, 72 slices, multiband acceleration factor 8, 2 mm isotropic voxel size, 104 × 104 matrix size, FOV 20.8 cm, 400 volumes per run). A fast 3D spoiled gradient recalled (FSPGR) T1-weighted sequence was used to acquire high-resolution structural images (0.8 mm isotropic voxel size, NEX=1, TE/TI=min full/1060 ms, flip angle 8°, FOV=25.6 cm, matrix=320 × 320, receiver bandwidth 31.25hz).

For the adolescent and adult cohorts, two separate 20 s spin-echo EPI sequences with opposing phase encoding directions were also acquired using the same matrix size, FOV and prescription to correct for susceptibility-induced distortions.

During all resting state functional scans, participants were presented with a white cross on a black screen and instructed to “Keep your eyes on the cross. Let your mind wander, relax, but please stay as still as you can. Try not to fall asleep.” Participants’ adherence to the instructions to remain awake, with eyes open, was monitored with an MR-compatible video camera. Heart rate and respiration during the functional scans were recorded continuously using a Biopac pulse oximeter and respiratory belt but were only of sufficient quality for analysis in the adult cohort (see Methods-Imaging data preprocessing and denoising).

### 2.4. Imaging data preprocessing and denoising

MRI data from all cohorts were preprocessed, denoised and analyzed in FSL (v5.0.10), FreeSurfer (v 5.3.0) and Matlab 2015b (Mathworks Inc., Natick, MA, USA) using SPM12 (Wellcome Trust Centre for Neuroimaging, University College London, UK), the CONN toolbox v17f, and custom Matlab code available upon request.

The structural image was converted from dicom to nifti format and was coregistered to the mean functional image, segmented and normalized to MNI space using non-linear registration and the default tissue probability maps included with SPM12. The white matter (WM) and CSF probability maps obtained from segmentation of the structural image for each individual subject were thresholded at 0.95 and eroded by 1 voxel. Due to high variability in ventricle size across adult participants, the template CSF map was used to extract CSF time courses (thresholded at 0.5 and eroded by 1 voxel). There was no overlap with gray matter voxels for the eroded WM and CSF masks for any participant. WM and CSF time courses were extracted from the thresholded and eroded masks using aCompCor (Behzadi et al., 2007) for subsequent nuisance regression (see below).

Functional images from the adult and adolescent cohorts were corrected for susceptibility-induced distortions using the two spin-echo EPI acquisitions with opposite phase encoding directions and FSL’s TOPUP tools (Smith et al., 2004). Functional images from all three cohorts were motion-corrected using rigid-body realignment as implemented in SPM12. The Artifact Detection Toolbox (ART, as installed with conn v17f) was used to identify outliers in the functional image time series from the resulting 6 motion parameters (3 translational and 3 rotational) that had frame-wise displacement (FD) >0.5 mm and/or changes in signal intensity that were greater than three standard deviations in the child/adolescent cohort. As oscillations due to respiration are prominent in motion parameters derived from multiband EPI realignment (Fair et al., 2018) and would result in unnecessary censoring of large segments of data in some participants, the thresholds to detect outliers in the adolescent and adult cohorts were more lenient than those used for standard resting state fMRI acquisitions with slower TRs (FD>0.9 mm and/or changes in signal intensity that were greater than five standard deviations). In order to ensure that none of our findings were due to differences in apparent motion between the ASD and TC groups, groups in each cohort were matched on RMSD (see Table 1) calculated from rigid-body realignment (of the raw data prior to TOPUP correction in case of the adolescent and adult cohort) parameters, and partial correlations controlling for RMSD were used when assessing brain-behavior relationships (Analysis 5).

Functional images were directly normalized to MNI space with the same non-linear registration as used for the structural images. Since all analyses were run on averaged voxel time series within pre-defined ROIs (see below), no prior smoothing was applied to the data. Voxel timeseries were normalized to percent signal change, with a time series mean of zero for each voxel. Normalization to percent signal change rather than z normalization was performed prior to DTW as recent studies have shown that the variability of the BOLD signal carries meaningful information about brain function (e.g. Bijsterbosch et al., 2017; Easson and McIntosh, 2019; Nomi et al., 2018, 2017) and the sensitivity of DTW to such differences might be an additional strength compared to PC (which is calculated on z normalized data). Band-pass filtering using a temporal filter of 0.008 to 0.08 Hz was carried out as part of the nuisance regression, which also included scrubbing of the motion outliers detected by the ART toolbox, and regression of the 6 motion parameters and their derivatives, as well as the first five PCA component time series derived from the eroded CSF and white matter masks. The residuals of the nuisance regression were then used for all subsequent functional connectivity analyses. Additionally, nuisance regression was repeated in the adult and child/adolescent cohort including the global signal (calculated as the average across all voxels in the brain) to test for the robustness of functional connectivity estimates to GSR (Analysis 1).

Physiological recordings during the fMRI scans were analyzed with the PhysIO toolbox (Kasper et al., 2017). Heart rate and respiratory measures (mean, standard deviation) were used to test the susceptibility of FC estimates to physiological noise in the adult cohort only (Analysis 5), as data quality particularly of the respiratory data was rarely of sufficient quality in children and adolescents due to poor equipment fit and frequent signal loss.

### 2.5. Functional connectivity estimates

Estimates of functional connectivity were derived from all cortical (91) and subcortical (14, excluding the brain stem) regions of interest (ROIs) of the Harvard Oxford atlas (Desikan et al., 2006). For Analysis 1 (Robustness to GSR) and Analysis 2 (Test-Retest Reliability) FC estimates were additionally extracted for 30 functionally defined network ROIs included in the CONN toolbox (v17f). These spanned 7 functional networks derived using Independent Component Analysis of resting state fMRI data from 497 HCP participants. For analysis 3, primary visual cortex (occipital pole) and cortical motor regions (precentral and postcentral gyrus) as defined in the Harvard-Oxford atlas were used. Time series were averaged across unsmoothed voxels in each ROI, and Pearson correlation between time series was calculated as the standard measure of FC, with Fisher z-transformed correlation coefficients used for all further analyses. Similarity of BOLD time series was further calculated between all ROI pairs using DTW. DTW warps two time series to optimally align while minimizing cost with its output reflecting a distance measure of the amount of warping required. This is achieved by searching the space of all possible pairwise distances between time points (constrained by the warping window size) for an optimal warping path. We refer the reader to Meszlényi et al. (2017b) for a technical description of DTW. Here, the dynamic time warping function implemented in MATLAB (https://www.mathworks.com/help/signal/ref/dtw.html) was used with a warping window size of 100 s, as was determined to be optimal for fMRI data by Meszlényi et al. (2017b). Further following the methods of Meszlényi et al. (2017b), DTW distances were then multiplied by −1 and demeaned to transform distance measures to similarity estimates that follow a normal distribution around 0, with values below 0 reflecting below average similarity of the time series and values above 0 reflecting above average similarity.

### 2.6. Analyses

#### 2.6.1. Analysis 1: robustness to motion and GSR

Given the substantial noise head motion can introduce into resting state fMRI data, we first tested the impact of motion on PC and DTW estimates of FC. Resting state BOLD time series were simulated for 20 participants and 25 distinct regions (excluding simulated CSF, WM and global components) using the SimTB toolbox implemented in Matlab (Erhardt et al., 2012). 180 volumes and 300 voxels were used for simulations of BOLD time series with a 2 s TR. As in Erhardt et al., 2012, rician noise was added to the data, with a uniform contrast-to-noise distribution ranging between 0.65 and 2 across participants. Motion was simulated for each participant with a maximum translation of 0.02 mm and a maximum rotation of 5°. PC and DTW were used to estimate FC between all regions before and after adding simulated motion to the time series. FC similarity was calculated by Pearson correlating FC estimates derived in the absence and presence of motion, with a higher correlation reflecting more robustness to motion. Additionally, the change in FC for each ROI pair was calculated (no motion - motion) and a paired-samples *t*-test was used to test for differences in FC similarity and change in FC (quantified by calculating the percent change of the FC difference using the no motion scan as the baseline for each ROI pair, taking the absolute value and then calculating the median across all ROI pairs; median absolute% change) for PC compared to DTW estimates of FC. The median change was chosen for this and subsequent analyses to minimize the impact of any potential outlier ROI pairs with extremely large FC changes and percent change was chosen in order to make the magnitude of FC changes for PC and DTW measures more directly comparable. To also test the effect of motion on FC estimates with real data, three fMRI scans were acquired from one participant (author AL, 33-year-old, female) while watching a 10 min. clip of Despicable Me (Vanderwal et al., 2019), two while instructed to stay as still as possible and one with intentional head motion. A natural stimulation “movie” design was chosen to minimize intra-subject variability introduced by different mental states that might be present during resting state fMRI and could decrease test-retest reliability. Imaging data were collected on a Siemens Prisma 3T at the SDSU Imaging Center (TR=2.4 s, TE=29 ms, flip angle 90°, 72 slices, 3 mm isotropic voxel size, 255 vol per run) and pre-processed using SPM12 and the Conn toolbox as described in Section 2.4. As for the simulated data, FC pattern similarity (across all cortical and subcortical Harvard Oxford ROIs) for scans with and without motion was calculated using Pearson correlation and changes in FC for each ROI pair were determined by subtracting FC estimates derived from the still and motion scans. A paired-samples *t*-test was used to test for differences in FC change between still and motion scans for PC compared to DTW estimates of FC.

To test for the impact of GSR on the robustness of FC estimates, **d**ata from the child/adolescent and adult cohort were denoised both with and without GSR and estimates of FC between Harvard-Oxford ROIs and the 30 network ROIs derived using PC and DTW were subtracted (noGSR – GSR) for each participant. This yielded a measure of how much FC estimates changed for each ROI pair when GSR was performed. A paired-samples *t*-test was performed to compare differences in the change of FC (median absolute% change across all ROI pairs) estimated using PC compared to DTW in each cohort separately. For the purpose of this analysis, ASD and TC cohorts were pooled to increase sample size. We hypothesized that FC would be more robust if estimated using DTW, thereby replicating findings by Meszlényi et al. (2017b).

#### 2.6.2. Analysis 2: test-retest reliability

In order to examine test-retest reliability when FC was estimated using PC compared to DTW, FC was estimated separately for the two consecutive resting state fMRI scans collected in each individual from the adult cohort. For each participant, Pearson correlation of FC matrices (Harvard-Oxford ROIs and the 30 network ROIs) from the two back-to-back scans acquired during the same MRI session was used to quantify the similarity of the FC pattern across scans, which served as a measure of test-retest reliability. ASD and TC participants were again analyzed as one group. Paired-samples *t*-tests were performed to compare test-retest reliability between PC and DTW estimates of FC. We hypothesized that DTW would show higher test-retest reliability.

#### 2.6.3. Analysis 3: sensitivity to brain function

While Meszlényi et al. (2017b) demonstrated increased robustness to GSR, higher test-retest reliability, and more accurate classification of gender when estimating FC using DTW compared to Pearson correlation, it remained unclear whether DTW was more sensitive to brain function or whether improved sensitivity was due to properties of the BOLD signal that could be independent of neural activity (e.g. temporal signal-to-noise differences due to head size, physiological differences between genders). We therefore assessed how FC between sensorimotor cortices changed with finger movements compared to a resting state condition, in order to test the sensitivity of FC estimates to brain function.

As part of a study on lexical decision making in adolescents with ASD and age, gender, and motion matched typically developing controls, we acquired both resting state and task fMRI (Child/Adolescent Cohort). Only the first task fMRI run was analyzed to match the duration of the resting state fMRI acquisition and only the first 300 volumes were analyzed for both the resting and task run. The task required that participants distinguish between animal and non-animal words, and to withhold a response if the stimulus was a pseudoword. All responses were made using a button box placed in the left hand (120 finger button responses, approximately every 2 s, interspersed with 114 null trials and 30 response suppression pseudoword trials). Performance accuracy was high in both diagnostic groups (mean 89.7% correct in the ASD and 93.6% correct in the TC group). Since the focus of this analysis was only on BOLD signal fluctuations related to button presses, we again analyzed the diagnostic groups together to increase sample size. We hypothesized that interhemispheric time series similarity between left and right motor cortices should decrease during task performance compared to resting state, given the frequent unilateral button presses required by the task (Kim et al., 2018; Moritz et al., 2000).

FC was estimated between left and right pre- and postcentral ROIs (as defined in the Harvard-Oxford atlas) using both PC and DTW. Since our main interest was in the change in FC during task performance compared to rest, the task design was not regressed out prior to FC estimation. We tested A) whether interhemispheric FC (precentral gyrus right – precentral gyrus left) differed during the task and resting state scans; and B) to ensure that the result was specific to the button press response and that FC was not generally lower during task performance, we further included intrahemispheric FC (precentral gyrus right – postcentral gyrus right) and interhemispheric connectivity between primary visual cortex (occipital pole) ROIs in 2 (rest, task) x 3 (ROI pairs) repeated measures ANOVAs. Additionally, change in FC between rest and task scans was determined by subtracting FC estimates (rest – task), and a paired-samples *t*-test examined whether the change in FC was larger when it was estimated using PC or DTW. We hypothesized that DTW would be more sensitive to differences in brain function between task and rsfMRI, and thus show a larger change in FC when the task was performed.

#### 2.6.4. Analysis 4: detection of group differences

To further test the sensitivity of PC compared to DTW estimates of FC to group differences or changes in brain function, we assessed which method more robustly detected alterations of FC in the child/adolescent and adult ASD cohorts, compared to age, gender and motion matched TC groups. FC estimates were derived using PC and DTW for all cortical (91) and subcortical (14) ROIs of the Harvard-Oxford atlas. First, *t*-tests were performed for FC of all ROI pairs to test for ASD-TC group differences. P-values from these *t*-tests (separately for PC and DTW estimates) were plotted in a histogram (Breheny et al., 2018) and the fraction of null hypotheses being incorrect were estimated as 1-*π*0 (Storey and Tibshirani, 2003). To illustrate which regions in the brain most strongly differentiated ASD and TC groups, the number of times an ROI was part of a pair with different (*p*<.05, uncorrected) FC in the ASD and TC groups was counted for each Harvard-Oxford ROI. Next, we tested whether classification of ASD was more accurate when using FC estimates derived using PC or DTW. PCA was used for feature reduction, retaining the top 5 PCA components and linear support-vector machine classification (with 15-fold cross-validation) conducted separately for PC and DTW FC estimates (1000 iterations). The top 5 PCA components were chosen as variance explained dropped steeply for additional components, with the lowest elbow for PC features observed at ~5 components. A Wilcoxon sign rank test was used to test for differences in classification accuracy. We hypothesized that DTW estimates of FC would be more sensitive to differences in FC in ASD.

#### 2.6.5. Analysis 5 relationship of FC estimates to behavior and physiology

Lastly, we tested whether PC or DTW estimates of FC showed more correlations with ASD symptom severity and executive function, as well as with physiological measures (respiration, heart rate) recorded during the fMRI scans that might confound functional connectivity estimates. For the child/adolescent cohort, ADOS-2 Total scores (ASD group only) and the BRIEF General Executive Composite (GEC), a continuously distributed measure within the ASD and TC groups, were Pearson correlated with FC estimates across all cortical Harvard-Oxford ROIs (controlling for motion during the resting state fMRI scans by using RMSD as a covariate). Additionally, the mean and standard deviation of respiratory volume per time (RVT), and mean and standard deviation of the heart rate (HR) recorded during the resting state scans in the adult cohort were also Pearson correlated with FC estimates (controlling for RMSD). Since sample size of the adult cohort was small and the adult ASD and TC groups did not show any differences in physiological measures, these correlations were conducted across groups to increase sample size. *P*-values from these correlations (separately for PC and DTW estimates) were again plotted in a histogram and the fraction of null hypotheses being incorrect was estimated as 1-*π*0, as in Analysis 4.

## 3. Results

### 3.1. Analysis 1: robustness to motion and GSR

**Fig. 2.**
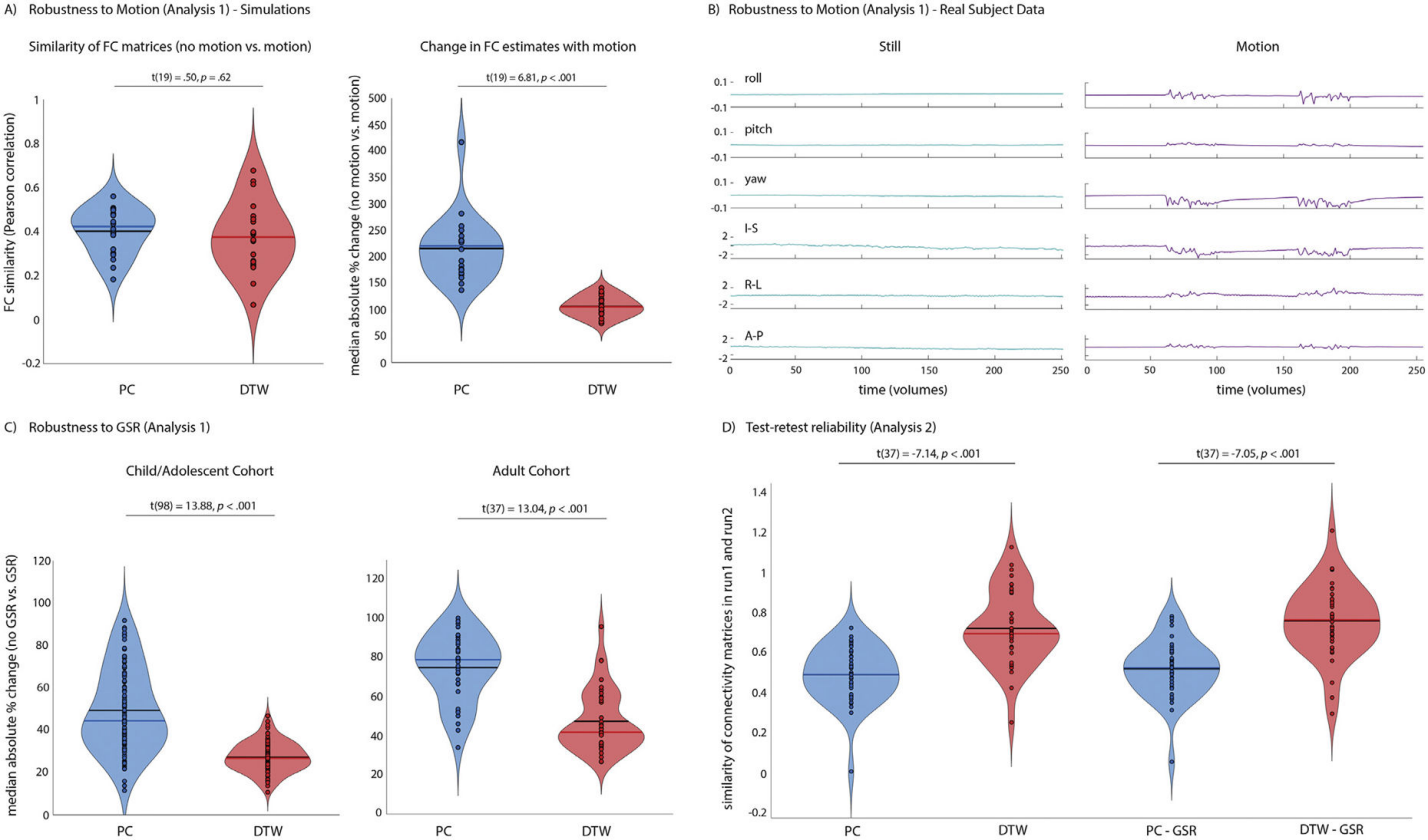
A) No significant difference in FC similarity between simulated data without and with added simulated motion was observed for DTW (red) compared to PC (blue) estimates of FC (left) indicating that DTW estimates do not seem to be more vulnerable to motion. In fact, changes in FC estimates for individual ROI pairs were significantly smaller in the presence of motion for DTW estimates (right). B) To test the impact of motion in real data, one participant (author AL) was scanned while watching the same movie clip from Despicable Me, while still and with intentional head motion. Realignment parameters are shown to illustrate the magnitude of motion. FC similarity between still and motion runs was reduced for PC estimates of FC (PC: *r* = 0.33, DTW: *r* = 0.47). C) Median absolute% change in FC estimated using Pearson correlation and DTW as a consequence of including vs. excluding the global signal in nuisance regression denoising of resting state fMRI data in the adult cohort, averaged across all cortical and subcortical Harvard-Oxford ROIs. D) Test-retest reliability of FC between two resting state fMRI scans acquired back-to-back during the same MRI session in each participant. Test-retest reliability was estimated as the similarity (Pearson correlation) of FC matrices across Harvard-Oxford ROIs.

Analysis 1 aimed to test the effect of motion on DTW compared to PC estimates of FC and to replicate previous findings of increased robustness to GSR reported by Meszlényi et al. (2017b). Simulations showed no significant differences in the impact of motion on the pattern of FC similarity for PC and DTW estimates of FC (*t*(19)=0.50, *p*=.62, Fig. 2A). When simulated motion was added to the time series, absolute changes in FC estimates (median absolute% change averaged across all ROI pairs), however, were larger when FC was quantified using PC (*t*(19)=6.81, *p*<.001, Fig. 2A; *t*(19)=2.9, *p*<.05 for mean absolute% change). When FC was compared for the same individual scanned while still and with intentional realistic motion (Fig. 1B), the similarity of FC patterns was higher for DTW estimates of FC for the two still scans (PC: *r* = 0.70, DTW: *r* = 0.87) and for still and motion scans (PC: *r* = 0.33, DTW: *r* = 0.47). There was no significant difference for the absolute% change in FC with motion for PC compared to DTW estimates of FC (*t*(5564)=0.95, *p*=.34).

GSR resulted in expected changes in FC, with the change in FC smaller (median absolute% change averaged across all ROI pairs) for DTW compared to PC estimates of FC for both the childhood/adolescent cohort (Harvard-Oxford ROIs: *t*(98)=13.88, *p*<.001; Network ROIs: *t*(98)=6.65, *p*<.001) and the adult cohort data (Harvard-Oxford ROIs: *t*(37)=13.04, *p*<.001; Network ROIs: *t*(37)=10.17, *p*<.001, Fig. 2C). Results were similar when using mean absolute% change (Harvard-Oxford ROIs; childhood/adolescent cohort: *t*(98)=4.09, *p*<.001; adult cohort (two outliers with means>3xSD excluded): *t*(35)=2.9, *p*<.01).

### 3.2. Analysis 2: test-retest reliability

We next tested whether previous results of increased test-retest reliability when using DTW to quantify FC replicated in a dataset that acquired two successive runs of rsfMRI. Test-retest reliability between the two consecutive rsfMRI scans acquired in the adult cohort was higher for DTW than PC estimates of FC (*t*(37)=2.57, *p*=.014, Fig. 2D), mirroring the results of the individual scanned for Analysis 1 described above. The difference was decreased when GSR was performed and was no longer significant (*t*(37)=1.88, *p*=.068).

### 3.3. Analysis 3: sensitivity to brain function

Functional connectivity was hypothesized to be reduced between left and right primary motor cortex during a task that required frequent unilateral left-hand button responses compared to resting state FC acquired from the same participants during the same fMRI session. A repeated-measures ANOVA tested whether changes in FC during the task were unique to primary motor cortex by including two control ROI pairs (intrahemispheric FC between right precentral and right postcentral gyrus and interhemispheric FC between primary visual cortices).

**Fig. 3.**
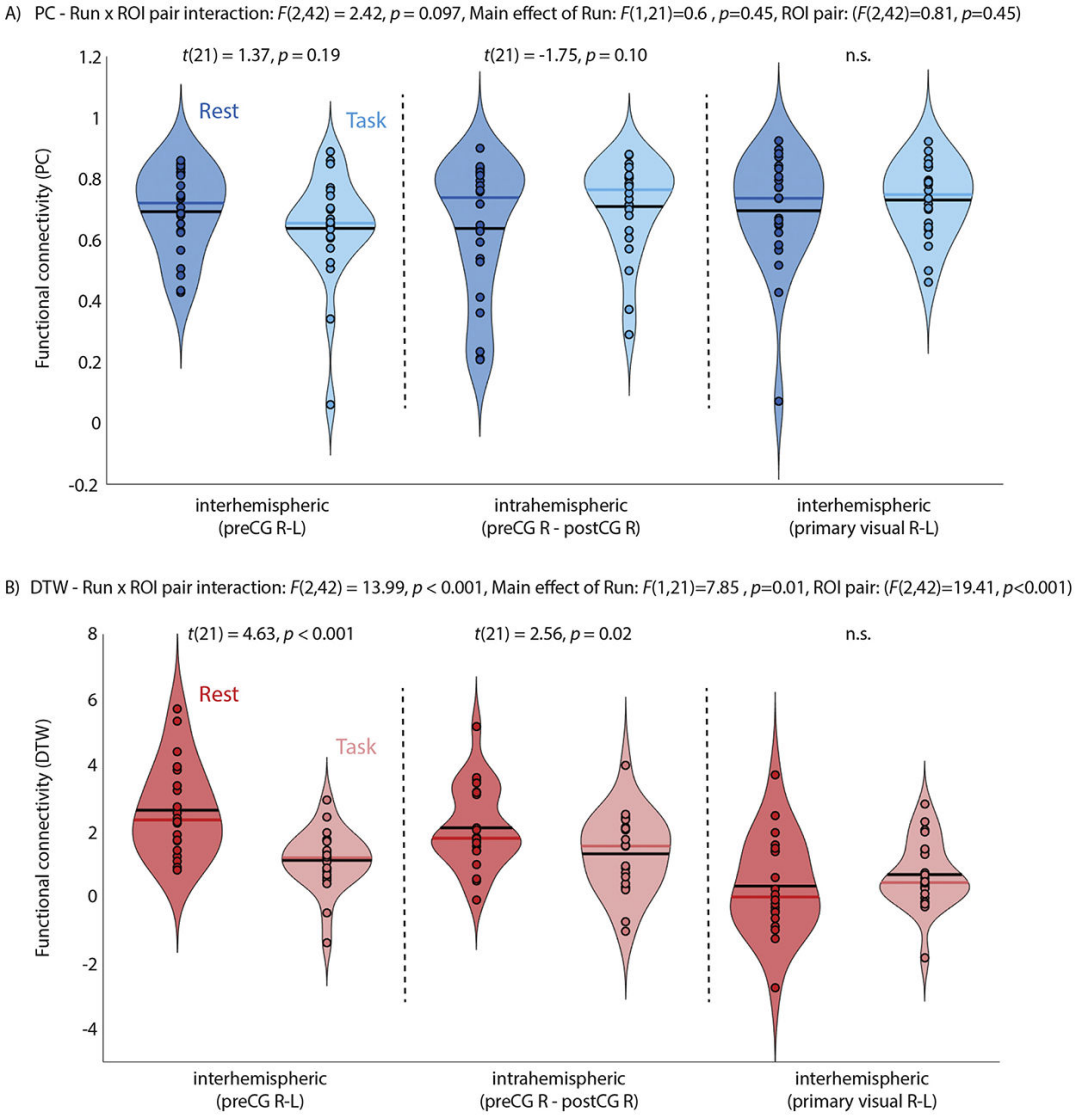
FC between cortical motor regions (pre- and post-central gyrus from the Harvard-Oxford atlas) during a resting state and a task fMRI in the same participants. A) Interhemispheric FC (preCG R-L) was reduced during the task compared to rest for DTW estimates of FC, as was expected given frequent button presses with the left index and middle finger during task fMRI. It did not differ for PC estimates of FC. Results of post-hoc pairwise paired-samples t-tests are reported for each comparison. B) Interhemispheric connectivity between left and right primary visual cortex was also estimated as a control and did not differ between rest and task fMRI for PC or DTW estimates of FC. Note, that for DTW estimates a value of 0 reflects average FC while positive values indicate above average time series similarity.

The main effects of run (rest, task: *F*(1,21)=0.6, *p*=.45) and ROI pair (*F*(2,42)=0.81, *p*=.45) and the run x ROI interaction (*F*(2,42)=2.47, *p*=.097) were not significant for PC estimates of FC, with neither inter-hemispheric (between homotopic left/right precentral gyrus ROIs) nor intrahemispheric (between right precentral and right postcentral gyrus ROIs) FC differing compared to resting state FC (Fig. 3A). For DTW estimates of FC, the main effects of run (*F*(1,21)=7.85, *p*=.01) and ROI pair (*F*(2,42)=19.42, *p*<.001) as well as the run x ROI pair interaction (*F*(2,42)=13.99, *p*<.001) were significant. Interhemispheric connectivity was reduced during task performance compared to rest (Fig. 3B), as hypothesized. Intrahemispheric connectivity was also reduced, but to a lesser extent. There were no significant differences for interhemispheric FC between left and right primary visual cortices during the resting state compared to task scan for PC or DTW estimates of FC. The median percent change in FC between the task and resting state scans across all motor ROIs was significantly greater for DTW than PC estimates (59.26% vs. 11.26%, respectively, *t*(21) = 5.72, *p* < .001).

### 3.4. Analysis 4: detection of group differences

**Fig. 4.**
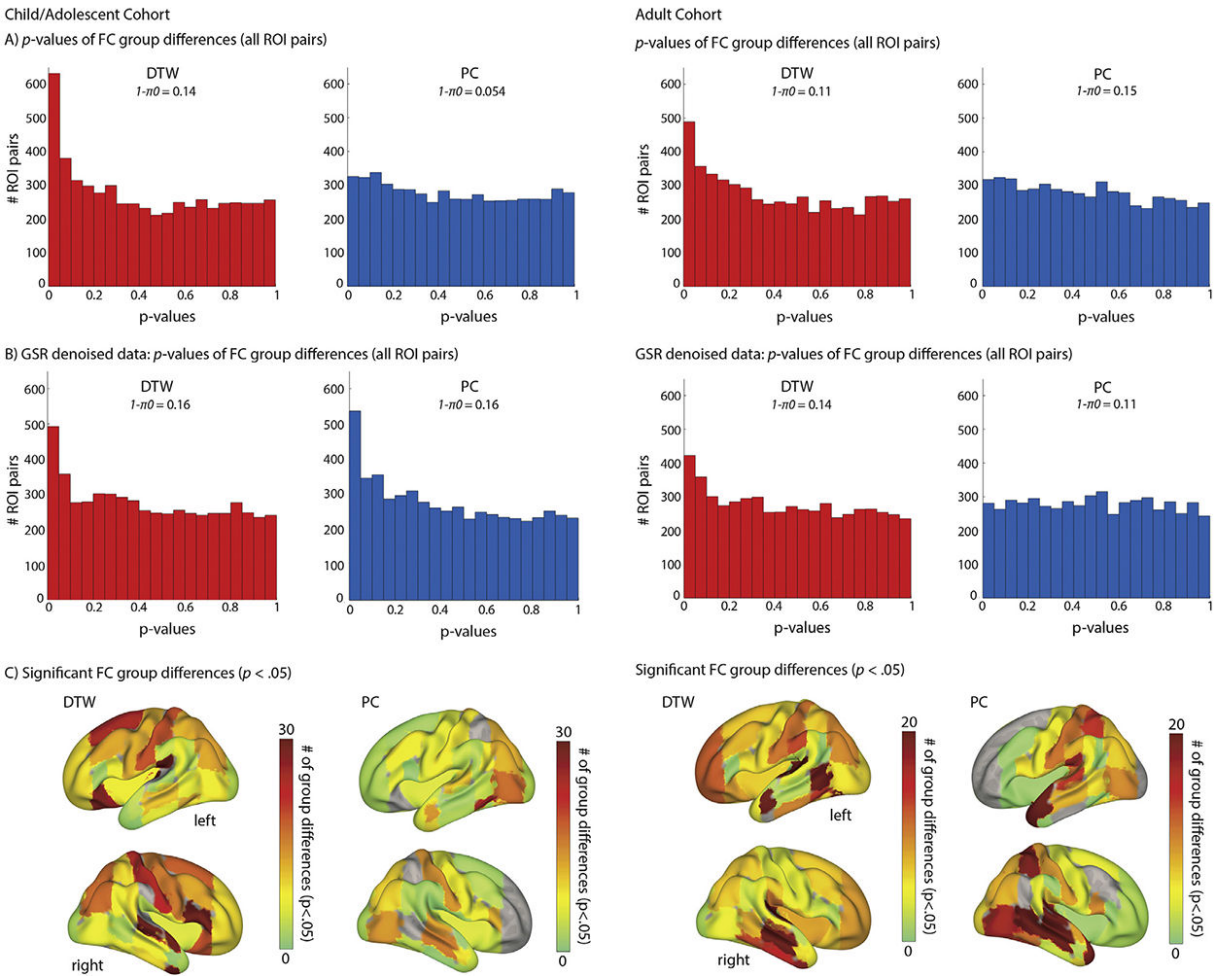
ASD-TC group differences detected for DTW and PC estimates of FC in the child/adolescent cohort (left) and adult cohort (right). Panels A (no GSR denoising) and B (GSR included during denoising) show enrichment for low p-values only for DTW estimates of FC, indicating evidence of a larger fraction of null hypotheses being false (note, that a uniform distribution is expected if all hypotheses are null). C) Shows the pattern of ROIs contributing most frequently to FC group differences (at an uncorrected significance threshold of *p*<.05, no GSR denoising).

A main goal of resting state fMRI in autism research is to identify group differences in FC. Analysis 4 tested whether atypical FC in ASD was more robustly detected when using DTW. More ROI pairs showed significant ASD-TD group differences (*p*<.05, uncorrected) when FC was estimated using DTW compared to PC in both the child/adolescent and adult cohorts (630/326 and 489/310 ROI pairs for DTW/PC estimates of FC for the child/adolescent and adult cohorts, respectively; Fig. 4A). More strikingly, low *p*-values were only enriched for DTW estimates of FC, with the fraction of null hypotheses estimated to be incorrect being substantially higher for DTW estimates. These analyses were repeated using data that had additionally been denoised using GSR (Fig. 4B). With GSR denoising, low p-values were also enriched for PC estimates of FC in the adolescent but not in the adult cohort. The frequency with which each ROI was part of a significant FC group difference is shown in Fig. 4C.

**Fig. 5.**
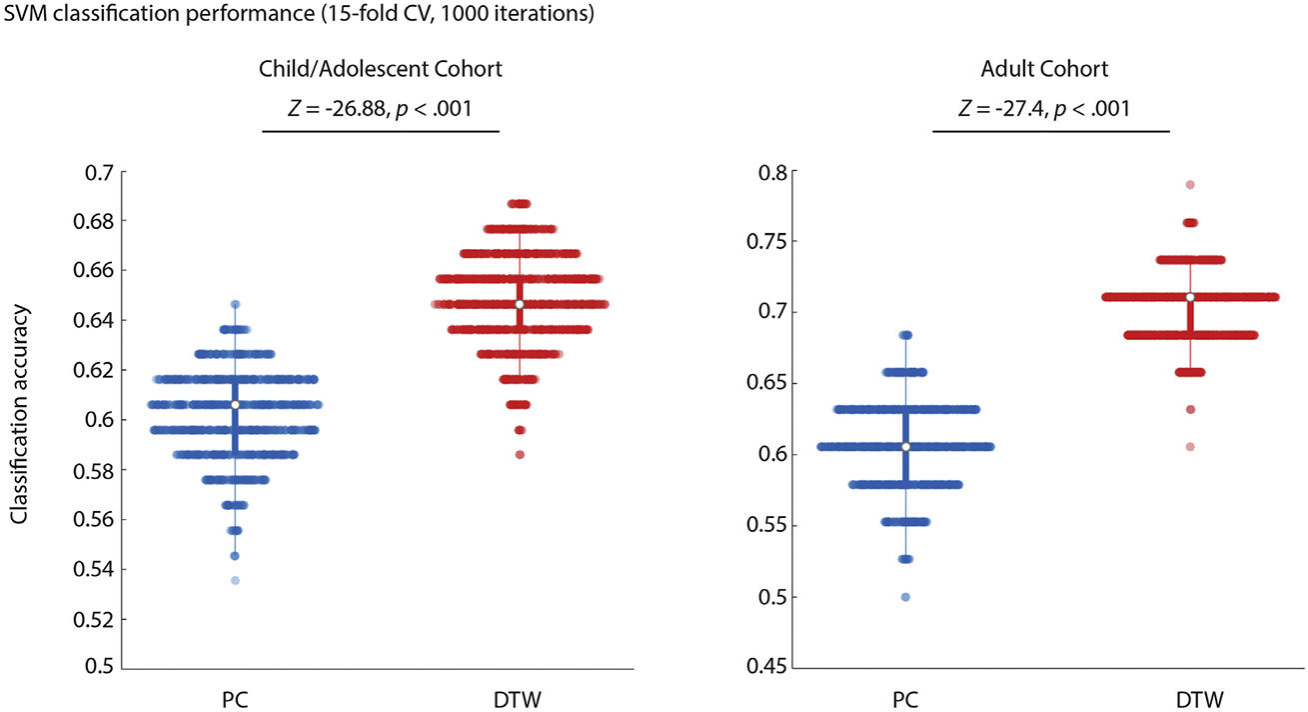
Linear SVM classification of diagnostic group (ASD/TC). The distribution of classification accuracy for 15-fold CV performed 1000 times using PC and DTW estimates of FC (top 5 PCA components used for classification) for the child/adolescent (left) and adult (right) cohorts.

Classification of diagnostic status achieved higher accuracy for DTW than for PC estimates of FC, in both the child/adolescent and adult cohort (Fig. 5).

### 3.5. Analysis 5: relationship of FC estimates to behavior and physiology

**Fig. 6.**
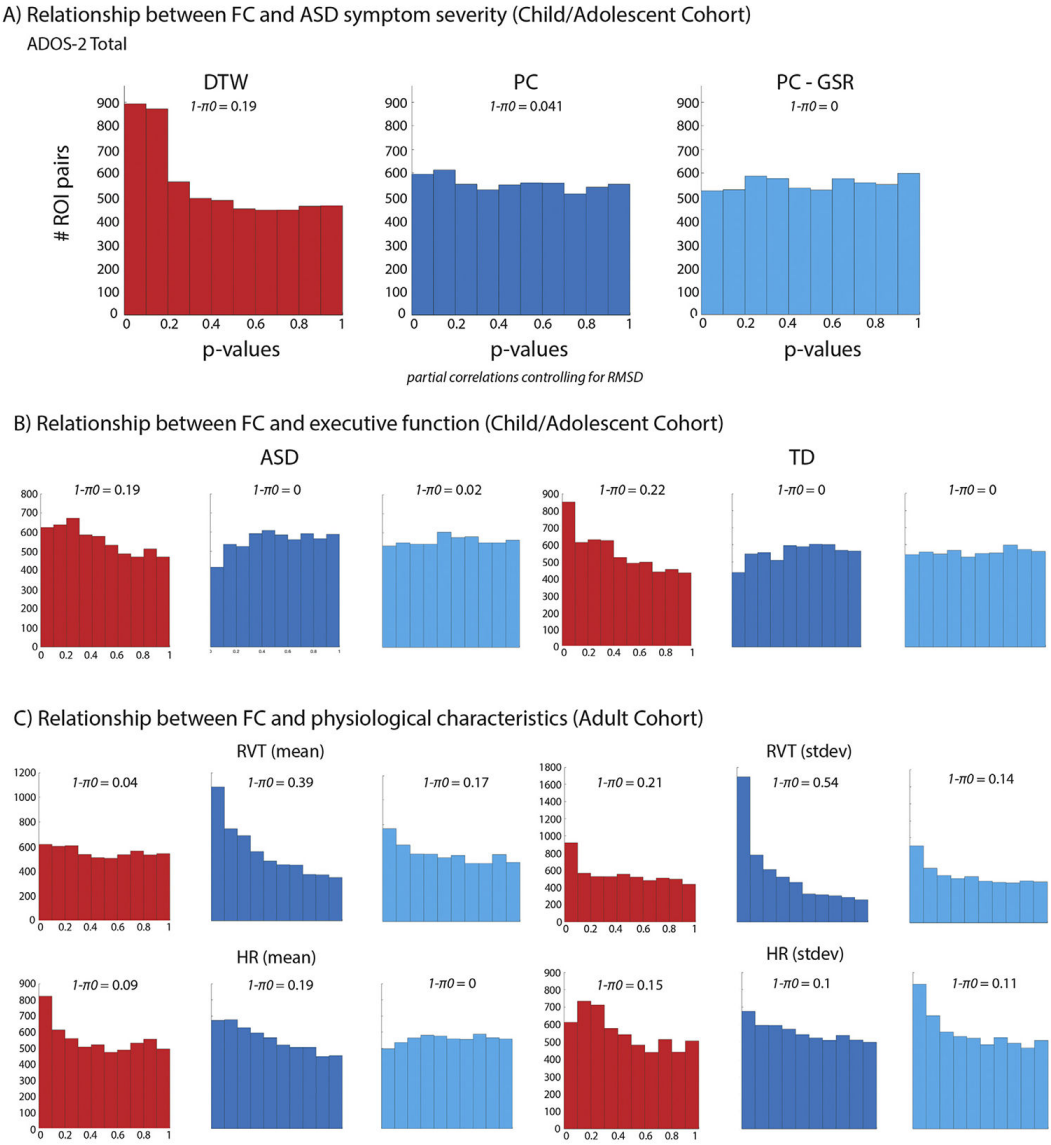
Enrichment for low *p*-values for the relationship (partial correlations controlling for RMSD) between FC and ASD symptom severity (A) and executive function (as measured by the BRIEF Global Executive Component) in the ASD and TD group separately (B) is only evident for DTW estimates of FC (Harvard-Oxford ROIs). For the relationship (partial correlations controlling for RMSD) between physiological measures (respiration and heart rate) and FC in the adult cohort results are partially reversed, with the number of null hypotheses rejected being generally higher for PC estimates of FC.

Lastly, we tested whether DTW or PC estimates of FC were more sensitive to brain-behavior relationships and potential physiological confounds. The association between FC estimates and ASD symptom severity was assessed in the child/adolescent cohort only, due to sample size limitations in other cohorts. Enrichment of low *p*-values for the correlation with ADOS-2 Total scores was only evident for DTW estimates of FC (Fig. 6A), with the fraction of null hypotheses rejected at 19% vs. 4.1% for DTW vs. PC estimates of FC, respectively. Similar results were observed in the ASD and TC groups for the BRIEF GEC (Fig. 6B). Only for DTW estimates of FC was there an indication of a relationship with executive function. Interestingly, this pattern was partially reversed for physiological variables, with a strong enrichment for small p-values for the association between PC FC and measures of respiration during the resting state scans (4% vs. 39% null hypotheses rejected for mean RVT, and 21% vs. 54% for RVT standard deviation, Fig. 6C top panel).

Results were more similar for measures of HR (9% vs. 19% null hypotheses rejected for mean HR for DTW compared to PC estimates of FC respectively, and 15% vs. 10% for HR standard deviation, Fig. 6C bottom panel). Since it is possible that PC estimates of FC would have shown a stronger relationship to behavior if GSR had been performed (Li et al., 2019b), the analyses were repeated using PC estimates of FC derived from data in which the global signal had been included during nuisance regression. The results largely mirrored those described above, with no enrichment for low *p*-values obvious for the relationship between PC FC estimates for ASD symptom severity or executive function (Fig. 6A/B). However, performing GSR reduced the associations between PC FC estimates and respiration (to 17% of null hypotheses rejected for mean RVT, and 14% for RVT standard deviation, Fig. 6C).

## 4. Discussion

Across five different analyses, our findings consistently suggest that dynamic time warping performs at superior levels, compared to Pearson correlation, in quantifying functional connectivity while also protecting more successfully from noise. We replicated increased robustness to GSR and higher test-retest reliability for DTW estimates of FC, as first reported by Meszlényi et al. (2017b). Additionally, we found that DTW FC was more sensitive to differences in brain function associated with task performance (compared to rest). Resting state fMRI and estimation of FC is frequently performed in clinical populations. Using DTW (compared to PC) estimates of FC, we observed improved classification accuracy of diagnostic status (ASD vs. TC) in cohorts of children and adolescents and of adults. Furthermore, we showed that group differences in FC were more frequently detected when using DTW rather than PC estimates of FC in two independent cohorts. Additionally, only DTW estimates of FC revealed robust associations with ASD symptom severity and executive function. Conversely, PC (but not DTW) estimates of FC were strongly related to respiration, suggesting that PC estimates are more sensitive to potential physiological confounds, especially when the global signal is not removed.

### 4.1. Effects of global, noise are smaller for DTW

Compared to PC estimates of FC, DTW estimates were less affected by GSR (Analysis 1). The smaller difference between DTW FC estimates from analyses with and without GSR implies that DTW is less sensitive to global noise, even when it is not explicitly removed. While it has been shown previously that removing the global signal from rsfMRI data improves the detection of relationships to behavior (Li et al., 2019b), this was not observed in the current analyses. Evidence for relationships between PC FC estimates and ASD symptom severity and executive function was low irrespective of performing GSR. Significant correlations between PC FC estimates and respiration, however, were substantially reduced when removing the global signal. Whether to use GSR during denoising of rsfMRI data remains highly controversial (Liu et al., 2017; Murphy and Fox, 2016; Power et al., 2017a; Uddin, 2017), due to concerns of introducing “artefactual negative correlations” (Murphy et al.,2009), distorting group differences (Gotts et al., 2013; Saad et al., 2012) or removing true neuronal signal (Li et al., 2019a; Schölvinck et al., 2010), and is therefore often avoided in resting state analyses. However, GSR also removes global noise (particularly from respiration) from rsfMRI data (Power et al., 2017a, 2017b; Power, 2019). The current analyses suggest that PC estimates of FC might be particularly vulnerable to confounds from respiration when they are not controlled for. Future studies need to investigate whether methods that might improve denoising (e.g. multi-echo ICA (Kundu et al., 2012; Power et al., 2018), RETROICOR (Glover et al., 2000), dynamic GSR (Erdoğan et al., 2016), or temporal ICA (Glasser et al., 2017)) may result in PC FC estimates that are more comparable to those from DTW in terms of differential sensitivity to brain function and noise. Pre-whitening would additionally account for temporal autocorrelations in fMRI time series, which is high particularly when analyzing fMRI data acquired with relatively high temporal resolution, and might further improve the test-retest reliability and sensitivity to brain function of Pearson correlation measures of time series similarity (Bright et al., 2017; Corbin et al., 2018).

### 4.2. DTW is robust to lag between brain regions

It remains unclear to what degree BOLD signal lag across the brain is neuronal rather than physiological. Some studies investigating BOLD lag structure, and related quasi-periodic patterns (QPPs), have made strong arguments in favor of a neuronal nature (Mitra et al., 2015, 2014; Thompson et al., 2014; Yousefi et al., 2018). However, physiological factors and differences in hemodynamics and blood arrival times clearly contribute to BOLD lag structure and FC patterns (Aso et al., 2017; Byrge and Kennedy, 2018; Chang and Glover, 2009; Chu et al., 2018; Golestani et al., 2017; Tong et al., 2018, 2015; Tong and Frederick, 2014; Wu and Marinazzo, 2016). For example, a recent study found that networks derived purely from respiratory and cardiac measures mimicked traditional functional networks derived from the BOLD signal using PC (Chen et al., 2019; also see Tong et al., 2015). Correcting for blood arrival times when regressing the global signal (“dynamic GSR”) also results in improved specificity and sensitivity of FC estimates (Erdoğan et al., 2016, also see Colenbier et al., 2019). In our study, PC estimates of FC were much more strongly associated with respiration than DTW estimates, potentially due to lag patterns introduced by respiration. In the presence of lag, Pearson correlation between two otherwise very similar time series will be low, making it difficult to separate lag from truly independent BOLD fluctuations. DTW on the other hand, will still identify similarity between the BOLD time series of brain regions with varying lag. This results in an estimate of FC that is not only easier to interpret but also less affected by potential physiological confounds. While beyond the scope of the current manuscript, further analyzing the shape of the warping path from which DTW estimates of BOLD time series similarity are calculated could offer additional information about how much lag or shape differences contributed to the FC estimate.

### 4.3. DTW is sensitive to BOLD signal amplitude

Lastly, while DTW is robust to lag and shape differences of the BOLD time series, it is sensitive to amplitude differences. The degree to which amplitude versus lag and shape differences influence FC estimates derived using DTW is currently not well understood. While the sensitivity of DTW to BOLD signal amplitude could benefit the detection of individual differences in brain function that are related to behavior (Bijsterbosch et al., 2017), it might also render it more sensitive to hardware differences (e.g. type of scanner or head coil) when pooling data from different sites and, thus, require careful standardization and harmonization (e.g. using tools such as ComBat, (Yu et al., 2018)). This will have to be carefully investigated prior to applying DTW to pooled data from different sites. While using Pearson correlations does not eliminate site differences and the need for data harmonization (Badhwar et al., 2019) its indifference to signal magnitude might make it slightly less sensitive to these confounds. Interestingly, however, this also means that PC can detect resting state networks even when BOLD sensitivity of an EPI sequence is low (e.g. because of a short echo time used to acquire fMRI data, Rane et al., 2014). The implications of this observation are difficult to unravel. While PC FC estimates are reduced at low TEs, resting state networks are still easily identifiable despite largely reduced BOLD sensitivity (Rane et al., 2014). This raises the question of whether the existence of these networks traditionally detected using Pearson correlation or ICA is predominantly driven by neuronal contributions to the BOLD response or by varying lag structure due to physiology and differences in blood arrival times (Tong et al., 2015).

In the absence of a gold standard for FC patterns across the human brain, it will be necessary to study and compare estimates of FC derived using multiple methods with different strengths and weaknesses. It is likely that DTW and PC estimates of FC are sensitive to different and partly complementary properties of functional brain organization. For example, Meszlényi et al. (2017a) found classification of mild amnestic cognitive impairment was improved when features derived from quantifying FC using Pearson correlation and DTW were combined. The results presented suggest that DTW is more sensitive to brain function and alterations of FC in ASDs than traditional Pearson correlation estimates. It remains to be determined how DTW estimates of FC relate to other measures of time series similarity (e.g. cross-correlation or cross-covariance) that can be derived from rsfMRI data. In a recent study comparing 9 different methods to estimate FC (Mohanty et al., 2020), DTW performed well and most methods resulted in FC estimates that were more closely related to behavior, and improved age classification. In network-level analyses, however, using correlation-based methods were shown to perform better than those sensitive to lag (Smith et al., 2011). It is possible that the appropriateness of the chosen time series similarity metric depends on the subsequent analysis methods, with Pearson correlation performing well in the context of network modeling but not when applied in analyses in which absolute FC magnitude matters (such as when testing for group differences in FC for specific ROI pairs, or when using pairwise FC estimates for classification). Ultimately, it is important to consider what characteristics render two time series similar and thus suggest involvement in the same underlying process prior to choosing a method to quantify FC from rsfMRI data. Interpretation of differences in FC magnitudes then need to be interpreted with the chosen method in mind.

### 4.4. Pearson correlation may impede detection of critical FC differences in ASD

The rsfMRI literature on ASD has almost exclusively relied on PC to estimate similarity of regional BOLD signal changes. This predominance of PC is broadly shared with FC research across different fields of clinical neuroscience and psychiatry (Rosazza and Minati, 2011). Our findings suggest that this reliance on PC may have significantly impeded FC research in the past decades. Specifically, with respect to ASD, we found that the alternative method of DTW afforded more extensive and robust detection of group differences (ASD vs. TC) in FC, better classification of diagnostic status, and improved detection of brain-behavior relations. These results have two implications for FC research on ASD, suggesting the possibility of both false positive and false negative findings. First, given evidence of greater sensitivity to noise in PC-based FC analyses and the possibility that differences in hemodynamic response and other physiological variables may be falsely interpreted as ‘atypical FC’, previous PC-based FC studies may have detected group differences between ASD and TC samples that do not truly reflect differences in neuronal activity and connectivity. Second, several of our findings suggest that PC-based FC studies may *fail* to detect group differences between ASD and TC cohorts as well as important brain behavior links (e.g., those related to symptom severity). This problem may be aggravated in studies with limited sample size and statistical power, which have been common in the literature, as relatively weak effect sizes in PC-based FC analyses may fail to reach arbitrary thresholds of ‘significance’. More generally, our findings suggest that research on FC in ASD (and beyond) may heavily benefit from alternative approaches that avoid the limitations of PC. Our results suggest that DTW may be such an alternative of choice.

### 4.5. Limitations

Limitations of our study include reliance on relatively small sample sizes in each cohort. It is reassuring, however, that previous results of increased robustness to GSR, higher test-retest reliability and improved classification accuracy reported by Meszlényi et al. (2017b) as well as Mohanty et al. (2020) replicated in our independent datasets. We are hopeful that the replication of these results and additional findings of improved classification accuracy of ASD diagnosis and relationship to brain function and behavior of DTW will result in more widespread adoption of DTW when quantifying FC, including replication in larger datasets. Our analyses were also restricted to two different parcellation schemes, with most analyses only carried out using the anatomically defined ROIs from the Harvard-Oxford atlas. We believe that an anatomical atlas is more appropriate for comparisons of PC with new FC quantification methods than a PC-derived parcellation that would conceivably bias comparisons. However, it will be necessary to test in future studies whether our results replicate across different brain parcellations. Particularly when studying clinical populations with possibly altered cortical differentiation (Dickie et al., 2018), individualized approaches to ROI definition might further improve sensitivity to group differences and brain-behavior relationships (Braga and Buckner, 2017; Gordon et al.,2017). Lastly, calculating time series similarity using DTW is computationally more intensive than linear methods such as PC (Silva et al., 2018). When running analyses in larger cohorts and when using fMRI data acquired with high temporal resolution or over a long period of time, parallelization is recommended.

### 4.6. Summary

While open questions remain, ours as well as a few previous studies (Meszlényi et al. 2017b, Mohanty et al., 2020) consistently suggest that non-linear methods such as DTW developed specifically for time series analyses should be more widely adopted in the analyses of functional connectivity derived from fMRI data. The use of different or additional metrics to quantify FC promises higher sensitivity to brain function, group differences and detection of brain-behavior relationships, addressing the low reliability of rsfMRI studies (Noble et al., 2019).

## Figures and Tables

**Table 1:** Demographics for the three cohorts. Means and SD as well as ranges are reported, and t-tests or chi-square tests (for sex) were carried out to assure that the ASD and TC groups were matched on age, in-scanner head motion (RMSD), and sex.

	Child/Adolescent Cohort			Adolescent Cohort			Adult Cohort		
	ASD *n* = 49	TC *n* = 50	*p*	ASD *n* = 11	TC *n* = 11	*p*	ASD *n* = 17	TC *n* = 19	*p*
**Age (years)**									
	13.65 (2.76) 7.4–17.6	13.32 (2.76) 8–18	.55	14.6 (2.1) 13–19	14.4 (1.3) 12–16	.72	49.7 (6.6) 40.4–60.6	50.4 (6.3) 40.6–60.9	.73
**Sex**									
# female	8	10	.64	2	1	.53	3	1	.24
**RMSD**									
rest	.06 (0.026) 0.017–0.11	.06 (0.03) **0**.017–0.14	.94	.08 (0.03) **0**.03–0.12	.06 (0.01) **0**.04–0.08	.14	.11 (0.05) **0**.04–0.22	.09 (0.05) **0**.05–0.19	.26
task	–	–	–	.08 (0.03) **0**.008–0.14	.06 (0.02) **0**.006–0.09	.18	–	–	–
**WASI-II**									
Verbal	103 (18) 59–147	108 (10) 87–133	.08	109 (13) 83–131	110 (17) 85–135	.83	101 (29) 45–160	116 (16) 85–144	.07
Non-Verbal	107 (19) 53–145	106 (13) 62–137	.72	112 (23) 75–156	107 (15) 80–128	.56	104 (24) 63–138	113 (11) 93–131	.13
Full-Scale	106 (18) 61–141	108 (11) 79–132	.50	112 (17) 77–136	110 (17) 88–135	.79	102 (24) 57–147	120 (11) 102–139	.01
**ADOS-2**									
SA	10.02 (3.9) 3–20	–	–	8.54 (2.88) 3–13	–	–	10.6 (3.8) 6–19	–	–
RRB	3.02 (1.7) 0–7	–	–	2.64 (2.54) 0–9	–	–	3.5 (2.0) 1–8	–	–
Total	13.3 (4.4) 5–24	–	–	10.3 (2.76) 7–16	–	–	14.2 (4.2) 8–23	–	–
